# Perinatal Outcome of Pemphigoid Gestationis: A Report of Three Cases and Review of the Literature

**DOI:** 10.7759/cureus.68582

**Published:** 2024-09-03

**Authors:** Ayako Inatomi, Daisuke Katsura, Shinsuke Tokoro, Shunichiro Tsuji, Takashi Murakami

**Affiliations:** 1 Department of Obstetrics and Gynecology, Shiga University of Medical Science, Otsu, JPN; 2 Department of Obstetrics and Gynecology, Shiga University of Medical Science, Ostu, JPN

**Keywords:** dermatoses of pregnancy, skin lesions, low birth weight, preterm birth, pemphigoid gestationis

## Abstract

Pemphigoid gestationis (PG) is a rare autoimmune blistering disorder that typically manifests during the second or third trimester of pregnancy. It is characterized by intensely pruritic urticarial plaques and blister formation, driven by an autoimmune response against the BP180 protein in the basement membrane. In this report, three cases of PG are presented, each illustrating distinct clinical courses and management strategies. The first case involves a 32-year-old primigravida at 31 weeks of gestation who presented with abdominal blisters that were unresponsive to topical steroids. Oral prednisone at a dosage of 15 mg was initiated at 33 weeks, leading to the resolution of the rash by 37 weeks. She subsequently delivered vaginally at 40 weeks. The second case concerns a 37-year-old multigravida who developed blisters on her limbs and abdomen at 27 weeks, which improved with the application of topical steroids. Due to a history of a previous cesarean section, she delivered via elective cesarean section at 38 weeks. The third case involves a 35-year-old multigravida who experienced fetal growth restriction starting from 29 weeks. She developed a mild erythematous, pruritic rash, and blisters at 33 weeks and required an emergency cesarean section at 33 weeks due to non-reassuring fetal status. The diagnosis of PG was confirmed postpartum. These cases underscore the clinical variability and potential complications associated with PG. They also suggest that the severity of PG's cutaneous manifestations may not directly correlate with pregnancy outcomes. Early detection and individualized management are crucial to optimizing both maternal and neonatal outcomes.

## Introduction

Pemphigoid gestationis (PG) is a rare and potentially severe autoimmune blistering disorder, occurring in approximately one in 50,000 pregnancies [1-3]. It characteristically presents during the second to third trimester but can also manifest during the immediate postpartum period. Clinically, PG is distinguished by the development of erythematous plaques and tense blisters, predominantly localized to the abdomen, particularly the umbilical region, as well as the buttocks and extremities. The diagnosis is typically established through histopathological examination of a skin biopsy, which reveals subepidermal blisters accompanied by neutrophilic and eosinophilic infiltration [2,4]. Furthermore, direct immunofluorescence (DIF) studies demonstrate the linear deposition of complement component C3 and immunoglobulins (often IgG) along the basement membrane zone, with the presence of circulating autoantibodies targeting BP180, a key hemidesmosomal protein [2,5].

The pathophysiology of PG involves a type II hypersensitivity reaction, where autoantibodies against BP180, also known as collagen XVII, initiate an inflammatory cascade leading to subepidermal blister formation. These autoantibodies bind to BP180, primarily located within the hemidesmosomes of the basement membrane, which results in immune complex formation and complement activation [6]. This process is detectable via direct immunofluorescence, which is crucial for differentiating PG from other pruritic dermatoses of pregnancy, such as pruritic urticarial papules and plaques of pregnancy (PUPPP), atopic dermatitis, and intrahepatic cholestasis of pregnancy. Importantly, unlike these other conditions, PG is associated with significant obstetric complications, including preterm birth and low birth weight, underscoring the importance of accurate diagnosis and management [1,7,8].

Recent studies suggest that the pathogenesis of PG may extend beyond the skin, involving the placenta and amniotic fluid. The detection of autoantibodies in these tissues implies a potential role in placental dysfunction, which may contribute to the adverse pregnancy outcomes observed in affected individuals [6,9]. There is evidence indicating that the presence and timing of blister formation correlate with pregnancy outcomes, highlighting the need for close monitoring and early intervention [10]. In this report, we present three cases of PG, detailing the clinical course and perinatal outcomes. These cases are discussed in the context of current literature to provide further insights into the management and prognosis of this challenging condition.

## Case presentation

Case 1

**Figure 1:**
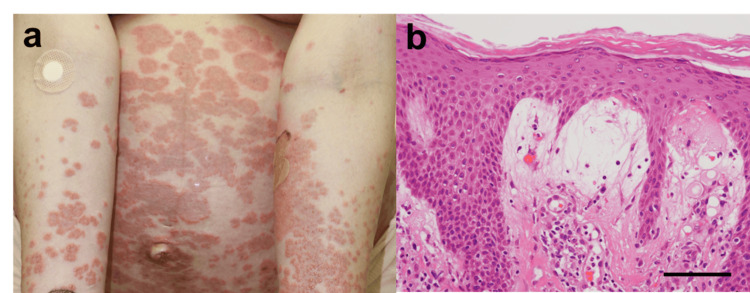
Clinical and Histopathological Findings in Case 1 (a) Clinical photograph of Case 1 showing erythema and blister formation around the umbilical area and extremities. (b) Hematoxylin and eosin (H&E)-stained section of the skin biopsy from Case 1, demonstrating subepidermal blistering with neutrophilic infiltration. Scale bar = 20 μm.

A 32-year-old nulliparous woman presented with a spontaneous pregnancy and was receiving routine antenatal care at a local obstetrics clinic. At 31 weeks of gestation, she developed erythema, pruritus, and vesicles localized to the periumbilical region, which subsequently disseminated to involve a larger portion of her body. At 33 weeks and three days of gestation, she was referred to our dermatology department due to persistent pruritus, erythema, and vesiculation (Figure 1a). Despite the initiation of topical corticosteroids, there was no significant improvement in her symptoms, necessitating hospitalization at 34 weeks and three days of gestation for comprehensive evaluation and management. Laboratory investigations at the time of admission revealed leukocytosis (WBC: 18,200/µl), anemia (Hb: 9.8 g/dl), platelet count of 223,000/µL, and a mildly elevated C-reactive protein (CRP) level of 0.64 mg/dl. Notably, anti-BP180 antibody titers were elevated at 159 U/ml. Fetal ultrasonography indicated an appropriate-for-gestational-age (AGA) fetus. On the day of admission, a skin biopsy was performed, which revealed subepidermal blistering on hematoxylin and eosin (H&E) staining (Figure 1b). Oral prednisolone was initiated at a dose of 15 mg/day starting from 34 weeks and three days of gestation. By 38 weeks, the skin lesions had begun to show signs of post-inflammatory hyperpigmentation, leading to a tapering of the prednisolone dose to 12.5 mg/day. The patient underwent spontaneous vaginal delivery at 40 weeks and two days of gestation. The neonate weighed 2,762 g, was appropriate for date (AFD), and had Apgar scores of 9 and 9 at 1 and 5 minutes, respectively. The umbilical artery blood pH was 7.266, with a base excess (BE) of -3 mmol/l. The neonate exhibited stable respiratory and circulatory function, and no neonatal cutaneous manifestations were observed. The postpartum course was uneventful, and no worsening of the skin lesions was observed after delivery. Both mother and child were discharged in good condition on postpartum day 5.

Case 2

**Figure 2:**
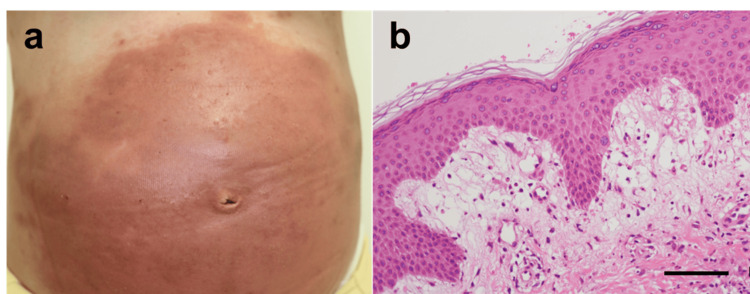
Clinical and Histopathological Observations in Case 2 (a) Clinical photograph of Case 2 illustrating extensive erythema and blistering around the umbilical region. (b) Hematoxylin and eosin (H&E)-stained section of the skin biopsy from Case 2, showing subepidermal blistering accompanied by neutrophilic infiltration. Scale bar = 20 μm.

A 36-year-old woman, gravida 2 para 1, conceived through egg donation due to a history of premature ovarian insufficiency. At 27 weeks of gestation, she developed pruritic papules on her extremities, prompting a consultation at our dermatology department at 29 weeks. She was diagnosed with pruritic eruption of pregnancy, for which topical corticosteroids were prescribed (Figure 2a). However, at 33 weeks and one day of gestation, vesicles emerged, accompanied by an exacerbation of symptoms. She was reevaluated in our department, and further diagnostic workup, including a skin biopsy and serological testing, was conducted. Anti-BP180 antibody titers were markedly elevated at 217 U/ml. Fetal ultrasonography confirmed normal fetal growth, consistent with an AGA fetus. Histopathological examination of the skin biopsy revealed subepidermal blistering (Figure 2b). Following the dermatological evaluation, a diagnosis of PG was established at 33 weeks based on histopathological findings. Although systemic corticosteroid therapy was considered, the decision was made to continue with topical corticosteroids due to the patient's gestational diabetes. Given her obstetric history of a prior cesarean section, a repeat cesarean delivery was planned at 38 weeks of gestation. The neonate weighed 3,176 g (AFD) and had Apgar scores of 8 and 9 at 1 and 5 minutes, respectively. The postpartum course was uneventful, with no signs of exacerbation in her skin condition after delivery. The neonate was discharged on day 6 postpartum, with no evidence of neonatal cutaneous involvement.

Case 3

**Figure 3:**
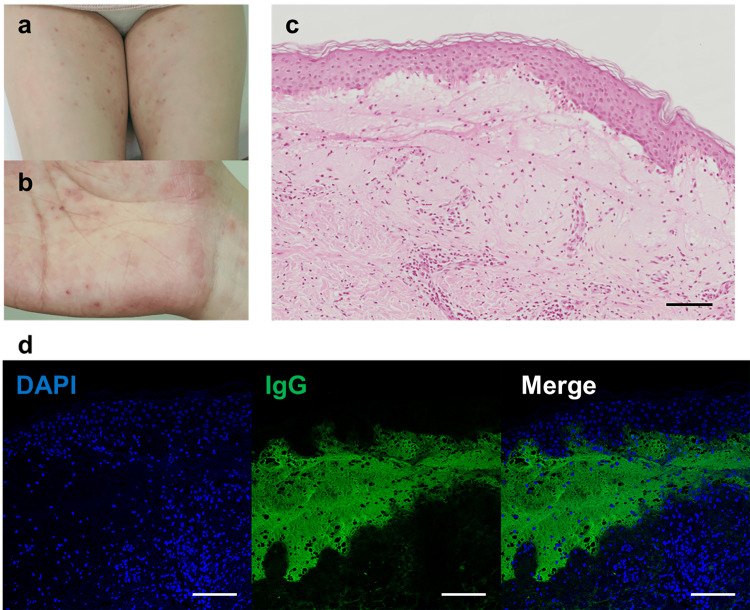
Clinical, Histopathological, and Immunofluorescence Analysis in Case 3 (a, b) Clinical photographs of Case 3 showing scattered erythema and blistering on the palms and thighs. (c) Hematoxylin and eosin (H&E)-stained section of the skin biopsy from Case 3, demonstrating subepidermal blister formation with neutrophilic infiltration. Scale bar = 20 μm. (d) Immunofluorescence staining of the skin biopsy from Case 3, revealing IgG deposition in the subepidermal blister area. Scale bar = 20 μm.

**Figure 4:**
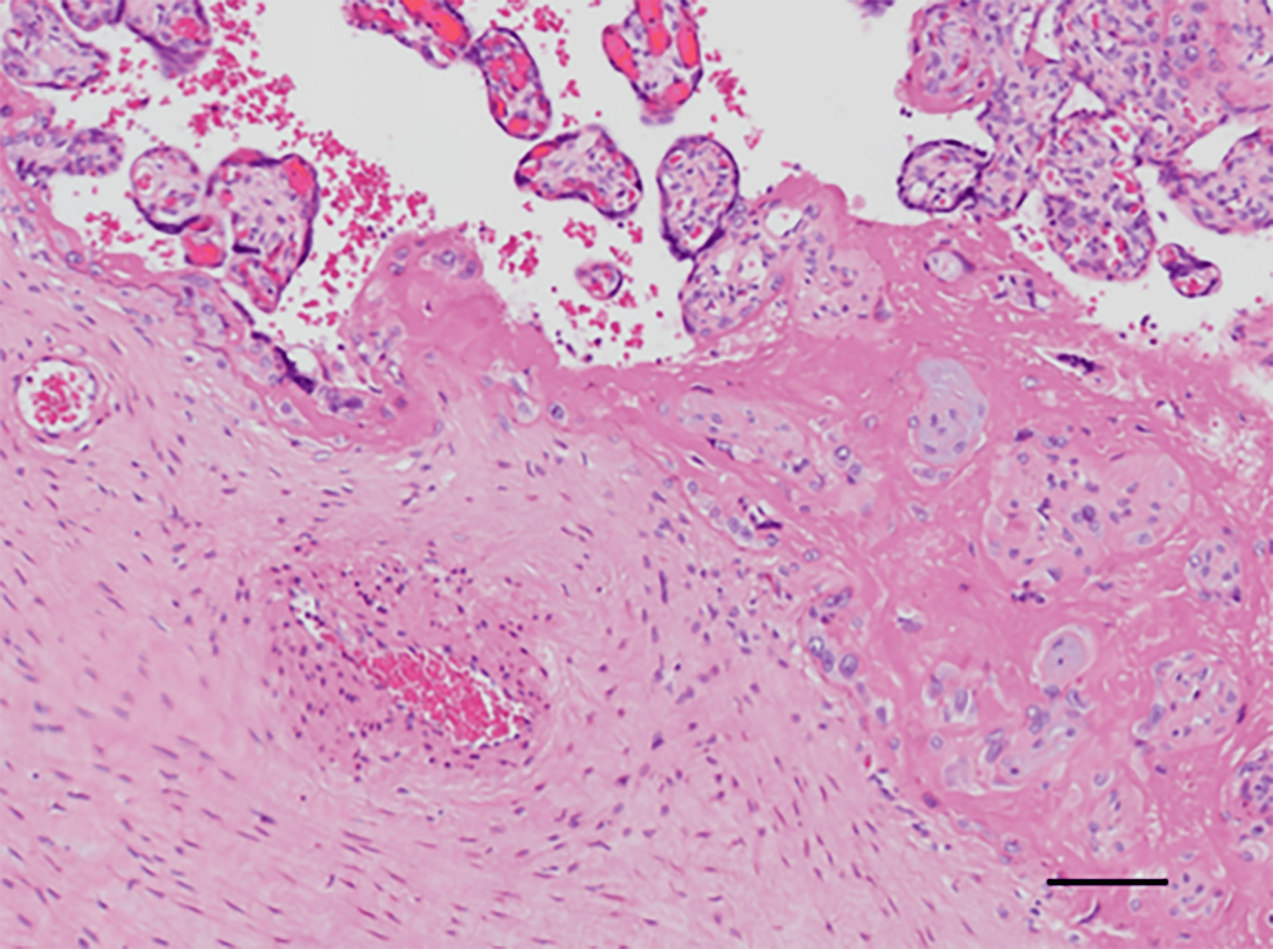
Histopathological Examination of the Placenta in Case 3 Hematoxylin and eosin (H&E)-stained section of the placenta from Case 3, showing degenerated villi with extensive fibrin deposition in the intervillous spaces. Scale bar = 20 μm.

**Figure 5:**
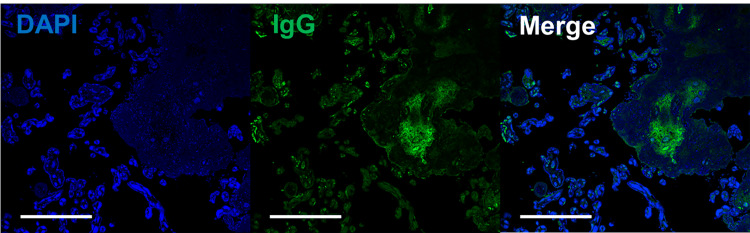
Immunofluorescence Findings in the Placenta from Case 3 Immunofluorescence staining of the placenta from Case 3, demonstrating IgG deposition around the blood vessels. Scale bar = 100 μm.

A 35-year-old woman, gravida 3 para 2, with a history of spontaneous conception, was receiving routine antenatal care at a local clinic. Around 29 weeks of gestation, fetal growth restriction (FGR) was identified. At 33 weeks and three days of gestation, the patient noted decreased fetal movements. Cardiotocography (CTG) revealed late decelerations, and oligohydramnios was also detected. Consequently, the patient was referred to our facility at 33 weeks and four days for further management, and she was admitted for inpatient care. On the same day, she developed erythema and pruritus affecting her extremities and buttocks. Laboratory evaluation demonstrated a WBC count of 6,500/µl, Hb of 12.2 g/dl, platelet count of 165,000/µL, and a mildly elevated CRP level of 0.64 mg/dl. Fetal ultrasonography confirmed the presence of FGR. During her inpatient stay, at 33 weeks and five days of gestation, the patient experienced a prolonged deceleration with a nadir of 80 bpm on CTG, prompting an emergency cesarean section due to non-reassuring fetal status (NRFS). The neonate weighed 1,412 g (low for date) and had Apgar scores of 4 and 9 at 1 and 5 minutes, respectively. The umbilical artery blood pH was 7.300, with a BE of -4.5 mmol/l. The neonate, being preterm and low birth weight, was admitted to the NICU, where the postnatal course was stable, leading to discharge on day 33 of life (corrected gestational age of 38 weeks and three days). No neonatal skin lesions were observed. Postoperatively, the mother developed scattered vesicles on her palms and persistent erythema with blisters on the periumbilical area, forearms, and thighs (Figures 3a, 3b). On postpartum day 5, she was re-evaluated by the dermatology department, where anti-BP180 antibody titers were found to be elevated at 92 U/ml. Topical corticosteroid therapy was initiated, leading to symptom improvement. Histopathological examination of a skin biopsy confirmed the presence of subepidermal blistering (Figure 3c). Placental pathology revealed degenerated villi with extensive fibrin deposition between them (Figure 4). Immunofluorescence staining demonstrated IgG deposition in the vesicular regions of the skin biopsy (Figure 3d), and similar IgG deposition was observed around the blood vessels in the placenta (Figure 5).

## Discussion

Pemphigoid gestationis is a rare autoimmune bullous dermatosis that predominantly manifests during the second trimester or later stages of pregnancy [1-3]. This disorder is characterized by intensely pruritic urticarial plaques and blister formation, which arise due to an autoimmune response targeting the basement membrane of the placenta [10]. Given the potential for significant maternal and fetal morbidity, accurate diagnosis and appropriate management of PG are critical [7]. This review aims to provide a comprehensive synthesis of the pathophysiology, clinical manifestations, therapeutic strategies, and complications associated with this condition, supplemented by a detailed report of cases encountered in our practice.

PG typically presents during the second and third trimesters, with the majority of cases occurring between the 20th and 34th weeks of gestation [1]. The peak incidence is observed in the later stages of pregnancy, likely reflecting the interplay between placental development and evolving maternal immune responses. Postpartum exacerbation of symptoms is also well-documented, with some cases showing rapid clinical deterioration following delivery. In a retrospective analysis of 61 cases, 21% were primiparous, while 38% had a history of PG. In addition, 5% of the pregnancies were complicated by maternal conditions that could adversely affect pregnancy outcomes, including preeclampsia and gestational diabetes [10]. Blisters were noted in 88% of cases, and preterm birth occurred in 34%. The onset of PG was most common in the second trimester (46%), followed by the first (31%) and third trimesters (23%). The study highlighted a correlation between blister presence, second-trimester onset, and an increased risk of preterm birth.

The diagnosis of PG is confirmed through clinical evaluation, histopathology, and direct immunofluorescence [2-4]. Histologically, PG is distinguished by subepidermal blistering with eosinophilic infiltration at the dermo-epidermal junction, a feature that aids in differentiating PG from other dermatoses [11]. Direct immunofluorescence reveals linear deposits of IgG and complement component C3 along the basement membrane, with BP180 (collagen XVII) being the primary antigenic target. The detection of circulating anti-BP180 antibodies, with reported sensitivities and specificities between 96% and 100%, further supports the diagnosis [10]. Although BP180 is expressed in the placenta from early gestation and has been identified in amniotic fluid, the pathophysiological significance of its deposition remains an area of ongoing investigation. Reports suggest that deposition of anti-BP180 antibodies within the placenta may lead to chronic placental dysfunction, contributing to adverse pregnancy outcomes. Despite morphological data on PG placentas being sparse, some studies have described the accumulation of C3, IgG, mild villitis, and clusters of immature fibrotic villi in PG-affected placentas [12]. These findings suggest a possible role for PG autoantibodies in disrupting placental architecture, potentially leading to obstetric complications such as preterm birth and fetal growth restriction. Notably, our series included a case (Case 3) that demonstrated severe fetal growth restriction, necessitating emergency cesarean delivery. This case highlights the critical need for vigilant fetal monitoring in PG-affected pregnancies. Immunopathological examination in Case 3 revealed IgG deposition in both skin lesions and the placenta, as illustrated in Figures 4 and 5, representing the first documented pathological evidence of such placental involvement in PG. Notably, in Case 3, which had the poorest pregnancy outcome, the severity of the rash was the least pronounced, and the elevation in serum anti-BP180 antibody levels was also mild. This observation suggests that the severity of pregnancy outcomes may not correlate with the extent of the rash or the level of anti-BP180 antibodies. The other two cases in our series had favorable outcomes, with full-term deliveries and no significant neonatal complications.

Numerous studies have documented a higher incidence of PG in multiparous women [1-3,10]. It is postulated that in women who have previously undergone sensitization of the immune system during earlier pregnancies, there is an increased likelihood of an immunological response against placental tissue in subsequent pregnancies. Repeated pregnancies and the hormonal milieu associated with them are hypothesized to modulate the immune system, potentially precipitating the recurrence or new onset of PG [4,13]. Furthermore, a history of PG is a known risk factor for recurrence in subsequent pregnancies, likely due to the persistence of memory immune responses. Consistent with these findings, two out of three cases in our series involved multiparous women.

In the differential diagnosis of pruritic dermatoses presenting in the second trimester, several conditions must be considered alongside PG, each with distinct clinical features and management approaches [1,7,8,14]. Pruritic Eruption of Pregnancy (PEP) typically presents as erythematous papules with intense pruritus, primarily affecting the extremities. It commonly manifests in the late third trimester and often resolves spontaneously postpartum. Unlike PG, PEP does not exhibit blister formation and is generally associated with minimal impact on maternal and fetal outcomes. Symptom management typically involves supportive therapy.

Pruritic Urticarial Papules and Plaques of Pregnancy (PUPPP) is another relatively common condition, particularly in multiple pregnancies. It typically arises in the third trimester, characterized by pruritic urticarial papules and plaques primarily involving the abdomen. Due to its clinical overlap with PG, early diagnosis can be challenging. However, PUPPP is generally benign, with no significant impact on pregnancy outcomes, and recurrence in subsequent pregnancies is rare. This is in contrast to PG, which has been linked to adverse outcomes such as preterm birth and low birth weight, with a notable risk of postpartum exacerbation and recurrence in subsequent pregnancies, underscoring the importance of accurate differential diagnosis.

Atopic dermatitis may also exacerbate during pregnancy, presenting with eczematous lesions and intense pruritus. In contrast to PG, atopic dermatitis typically does not form blisters, although symptoms may worsen due to hormonal and immune fluctuations during pregnancy.

Drug-induced dermatoses should also be considered, as certain medications can induce hypersensitivity reactions characterized by erythema, rashes, and in some cases, blister formation. A thorough review of the patient's medication history is essential for diagnosis, with improvement upon discontinuation of the offending agent being a key diagnostic indicator.

Therapeutic management of PG is dictated by the severity of symptoms. Mild cases may be effectively managed with topical corticosteroids and antihistamines to alleviate pruritus and prevent lesion progression [15]. However, moderate to severe cases typically require systemic corticosteroids (e.g., prednisolone) to achieve adequate disease control [16]. While systemic corticosteroids are effective in suppressing inflammation, their long-term use is associated with potential side effects, necessitating careful monitoring [17]. In refractory cases, immunosuppressive agents such as cyclosporine or mycophenolate mofetil may be considered as adjunctive therapy, particularly when corticosteroids alone are insufficient. Additionally, antihistamines may provide symptomatic relief of pruritus [18]. Notably, studies have found no significant association between systemic corticosteroid use and adverse pregnancy outcomes, including birth weight, suggesting that with appropriate dosing and monitoring, these therapies can be safely administered during pregnancy [2].

## Conclusions

Although PG is a rare condition, its potential to cause significant obstetric complications such as preterm birth and fetal growth restriction warrants prompt recognition and intervention. When confronted with severe pruritus, widespread erythema, and blistering during pregnancy, PG should be considered in the differential diagnosis, and timely diagnosis and treatment should be pursued. Effective management of PG can lead to improved maternal and fetal outcomes, emphasizing the importance of clinician awareness of its characteristic features and diagnostic strategies.
